# Vertical variations in microbial diversity, composition, and interactions in freshwater lake sediments on the Tibetan plateau

**DOI:** 10.3389/fmicb.2023.1118892

**Published:** 2023-03-08

**Authors:** Xinshu Zhu, Yongcui Deng, Tao Huang, Cheng Han, Lei Chen, Zhigang Zhang, Keshao Liu, Yongqin Liu, Changchun Huang

**Affiliations:** ^1^School of Geography, Nanjing Normal University, Nanjing, China; ^2^Jiangsu Center for Collaborative Innovation in Geographical Information Resource Development and Application, Nanjing, China; ^3^State Key Laboratory of Tibetan Plateau Earth System, Resources and Environment (TPESRE), Institute of Tibetan Plateau Research, Chinese Academy of Sciences, Beijing, China; ^4^Center for the Pan-Third Pole Environment, Lanzhou University, Lanzhou, China

**Keywords:** lake sediment, microbial community, β-diversity, co-occurrence network, Tibetan plateau

## Abstract

Microbial communities in freshwater lake sediments exhibit a distinct depth-dependent variability. Further exploration is required to understand their biodiversity pattern and microbial interactions in vertical sediments. In this study, sediment cores from two freshwater lakes, Mugecuo (MGC) and Cuopu (CP), on the Tibetan plateau were sampled and subsequently sliced into layers at a depth of every centimeter or half a centimeter. Amplicon sequencing was used to analyze the composition, diversity, and interaction of microbial communities. Results showed that sediment samples of both lakes could be clustered into two groups at a sediment depth of about 20 cm, with obvious shifts in microbial community compositions. In lake MGC, the richness component dominated β-diversity and increased with depth, indicating that the microbial communities in the deep layer of MGC was selected from the surface layer. Conversely, the replacement component dominated β-diversity in CP, implying a high turnover rate in the surface layer and inactive seed banks with a high variety in the deep layer. A co-occurrence network analysis showed that negative microbial interactions were prevalent in the surface layers with high nutrient concentrations, while positive microbial interactions were more common in the deep layers with low nutrient concentrations, suggesting that microbial interactions are influenced by nutrient conditions in the vertical sediments. Additionally, the results highlight the significant contributions of abundant and rare taxa to microbial interactions and vertical fluctuations of β-diversity, respectively. Overall, this work deepens our understanding of patterns of microbial interactions and vertical fluctuation in β-diversity in lake sediment columns, particularly in freshwater lake sediments from the Tibetan plateau.

## Introduction

Lake sediment is an essential global organic carbon sink (Dean and Gorham, 1998), formed by the interaction of deposition and decomposition of organic matter (OM) in the overlying water (Ambrosetti et al., 2003). Like marine sediments, as the depth increases, the contribution of refractory organic matter also increases while the availability of electron acceptors decrease (Ambrosetti et al., 2003; Wurzbacher et al., 2017; D'Hondt et al., 2019), which may lead to changes in the microbial communities in the lake sediments (Rissanen et al., 2019). Environmental factors such as nutrients, salinity, and pH, which vary with depth, also impact the microbial community structure in sediment environments (Edlund et al., 2006; Nelson et al., 2007; Zhao et al., 2011). Microorganisms in sediment play a crucial role in OM mineralization and in nutrient cycling and energy transfer processes (Mason et al., 2009). Understanding the microbial communities present in vertical lake sediments enables us to investigate the depth-related changes in ecosystem processes.

The β-diversity, as the extent of change in community composition, was first proposed in 1960 (Whittaker, 1960). Previous studies have found that β-diversity could reflect nestedness and spatial turnover (Harrison et al., 1992; Baselga, 2007, 2010). Nestedness, also named richness difference, reflects the change in the relative abundance of ever-present taxa (Legendre, 2014). Spatial turnover, also called replacement, means the extent of replacement for different species without the influence of richness difference (Baselga, 2010). Partitioning β-diversity into nestedness and turnover components could reveal ecological processes for species composition variation in the sediment core. For example, a nested microbial community selected from the lake surface layer was found in the lake’s deep layer (Vuillemin et al., 2018), indicating that microbial community variation is mainly driven by the environment filter effect. However, a high replacement effect was also found to be dominant in the upper sediments of lakes, indicating the impact of cellular turnover and random appearances on microbial communities (Wurzbacher et al., 2017).

Microorganisms interact with each other in different ways. Some strains may cooperate and rely heavily on their allies, while others may compete with each other for basic finite resources like nutrients, light, or territory (Bauer et al., 2018). The nutrient concentration has been found to affect bacterial interactions in pure culture studies and *in situ* habitats (Khan et al., 2018; Velez et al., 2018; Bergk Pinto et al., 2019; Hester et al., 2019). Those studies suggested that positive interactions predominate in environments with limited nutrients, while negative interactions predominate in nutrient-rich environments. Previous research on marine and lake surface sediment has shown that negative microbial interactions increase with nutrient supplementation (Dai et al., 2022; Zhang et al., 2022). However, the extent to which microbial interactions change with depth in vertical lake sediment with varied nutrient concentrations remains unclear.

Microbial communities generally consist of a few abundant and many rare species (Logares et al., 2014). Abundant and rare species are defined based on their relative abundance with a specific cutoff level. For instance, species with a relative abundance higher than 0.1% are defined as abundant (Jiao and Lu, 2020). Besides, the frequency of species occurrence is also taken into consideration (Dai et al., 2022). Abundant and rare taxa show different characteristics and ecological roles (Pedros-Alio, 2012; Logares et al., 2014). Abundant taxa play a crucial role in biomass, carbon flow, and nutrient cycling, while rare taxa are essential for biodiversity and redundant functions (Pedros-Alio, 2012; Debroas et al., 2015). Previous research has revealed differences in the distribution patterns and functional traits of abundant and rare subcommunities in various environments (Zhang X. et al., 2021; Ren et al., 2022). Therefore, incorporating both abundant and rare subcommunities can provide a more comprehensive evaluation of the impact of microbial communities on vertical lake sediments.

High-altitude lakes, such as those on the Tibetan plateau, are more sensitive to the impacts of human activities and climatic change due to their limited water exchange compared with low-altitude lakes (Sala et al., 2000; Messerli et al., 2004). Freshwater lakes make up a significant portion of the total lake area on the Tibetan plateau (Zheng et al., 1993) and play important roles in ecosystem balance (Liu et al., 2009). Most freshwater sediments have many different environmental niches, even on a millimeter-scale (Spring et al., 2000). The sediment microbial community in these lakes can serve as an indicator of ecosystem health and environmental conditions (Dai et al., 2016; Zhang et al., 2019). Further studies on the vertical β-diversity and microbial interactions could deepen our understanding of the potential ecological mechanism governing microbial community in freshwater lake sediments. In this study, we obtained sediment cores from two freshwater lakes, Mugecuo Lake and Cuopu Lake. Vertical variations in microbial communities were analyzed through quantitative PCR (qPCR) and high-throughput 16S rRNA gene sequencing techniques. Our objective is to examine the following: (1) The relationship between microbial community composition and potential function with depth in freshwater lake sediments, (2) The change in vertical beta diversity with depth, and (3) The shifts in microbial interactions with sediment depth and nutrient concentration.

## Materials and methods

### Study site and sampling

Mugecuo Lake (MGC, 101.8414 E–101.8708 E, 30.1626 N–30.1483 N) and Cuopu Lake (CP, 99.3344E–99.6458E, 30.3731 N–30.6353 N) are freshwater lakes located on the eastern Tibetan plateau (Supplementary Figure S1). MGC and CP have altitudes of 3,793 and 4,230 m, water area of 4 km^2^ and 0.9 m^2^, and maximum water depth of 70 and 15 m, respectively, (Ma et al., 2019; Jiang et al., 2020). In October 2017, lake sediment cores from MGC lake (30.1492 N, 101.8531E) and CP lake (30.4917 N, 99.5442E) were sampled using a Gravity sampler. The water depth at the sampling location at MGC was ~35 m and at LC was ~14 m. The collected sediment cores of were 38.5 and 45 cm long for MGC and CP, respectively. The cores were sliced into 76 and 45 samples at 0.5 or 1 cm intervals for MGC and CP, respectively. Each sample was divided into two subsamples—one for analyzing soil physicochemical index and the other for molecular biological analysis.

### Sediment properties measurement

The sediment samples were analyzed for various properties, including total organic carbon (TOC), total nitrogen (TN), ammonia nitrogen (NH_4_^+^-N), nitrate nitrogen (NO_3_^−^-N), and inorganic phosphorous (IP). The TOC was detected using a TOC analyzer (TOC-L&SSM, Shimadzu Corp, Japan) with the dry combustion method. The TN was measured using an ultraviolet spectrophotometer (UV 3600, Shimadzu Corp, Japan) with the sulfate digestion method. The NH_4_^+^-N and nitrate nitrogen NO_3_^−^-N were measured using a continuous flow analyzer (Skalar, Breda, and Netherlands). The IP was measured following the Standards Measurements and Testing (SMT) protocol (Wu et al., 2018).

In addition, the specific activity of ^226^Ra and ^210^Pb was measured using a high-resolution high purity Germanium (HPGe) γ-spectrometer (EG&GORTEC, GWL-120-15, United States). The age of the sediment deposition was calculated using the constant rate of supply (CRS) of the ^210^Pb model (Sanchez-Cabeza and Ruiz-Fernández, 2012).


$$t=\frac{1}{\lambda }\times \ln\left(\frac{A_{h}}{A_{0}}\right)$$

Where *λ* is the decay constant of ^210^Pb (*λ* = 0.03114/a), *A_h_* is the ^210^Pb_ex_ activity of each sedimentary layer, and *A_0_* is the total activity of ^210^Pb in the sedimentary core.

### DNA extraction, quantitative PCR, and Illumina sequencing

To minimize the risk of DNA contamination, all the molecular experiments were conducted in a sterile environment using 75% ethanol-cleaned and UV sterilized laboratory tools. The total DNA of lake sediments was extracted using the FastDNA SPIN Kit (MP Biomedicals, United States), and its concentration was quantified using a Nanodrop ultraviolet spectrophotometer (Thermo Scientific, Wilmington, United States). The extracted DNA was stored at −20°C until further analysis.

The abundance of 16S rRNA gene in the sediment samples was detected by quantitative PCR (qPCR) using 515F (5′-GTGCCAG CMGCCGCGGTAA-3′) and 907R (5′-CCGYCAATTYMTTTRA GTTT-3′) primer pairs (Kolb et al., 2003). The qPCR reactions were performed on a CFX96 Real-Time System (BioRad, Laboratories Inc., United States), with an initial denaturation (95°C, 3 min) followed by 40 cycles of denaturation at 95°C for 10 s, annealing at 55°C for 20 s, and extension at 72°C for 20 s. The qPCR reaction volume included 2 μL of template DNA, 0.4 μL of each primer (10 μM), 10 μL of SYBR Green and ROX mixture (2×, Accurate Biotechnology Co., Hunan, China), and 7.2 μL of sterile water. Melting curve analysis was used to test the specificity of PCR products. The *R*^2^ and amplification efficiency of the standard curve were 0.996 and 99%, respectively.

PCR amplicon was carried out targeting 16S rRNA gene V4–V5 region using primer pairs 515F and 907R (Kolb et al., 2003). The PCR was performed in a 50 μL reaction volume, including 2 μL of the template DNA, 1 μL of each primer (10 μM), 25 μL of EasyTaq ® PCR SuperMix (2×), and 22 μL of sterile water. The PCR cycling was performed on a S100™ Thermal Cycler (BioRad, Laboratories Inc., United States), with an initial denaturation (95°C, 1 min) followed by 28 cycles (denaturation at 95°C for 20 s, annealing at 55°C for 20 s, and extension at 72°C for 30 s), followed by a final extension step (72°C for 10 min). The PCR products were purified with Omega Bio-Tek E.Z.N.A.® Cycle Pure Kit and quantified using Nanodrop ultraviolet spectrophotometer (Thermo Scientific, Wilmington, United States). Finally, the purified DNA was sequenced on an Illumina HiSeq2500 platform at Magigene Biotechnology Co. Ltd. (Guangzhou, China).

### Sequencing analysis

The 16S rRNA raw data were processed using USEARCH (v10.0) and Mothur (v1.45.2) to perform the sequencing analysis. Firstly, the paired reads of raw sequences were merged using USEARCH’s-fastq_mergepairs command, and the forward and reverse primers were removed. After filtering quality, sequences with a maximum expected error greater than one were excluded. The Operational taxonomic units (OTUs) were generated by clustering the sequence at 97% sequence similarity using USEARCH’s-cluster_otus command. The obtained OTU table was then classified using the command Mothur’s classify. Seqs command, using the SILVA 138 reference database. The alpha diversity indices, including Chao1, Shannon, Simpson, and Richness, were calculated in USEARCH using the-alpha_div command based on the normalized OTU table. All the 16S rRNA sequences have been deposited in the SRA database of the national center for biotechnology information (NCBI) with the accession number PRJNA876099.

### Statistical analyses

To assess the microbial community by considering subcommunities with different relative abundance, we defined the “abundant” OTUs as the OTUs with relative abundance >0.1% in more than 50% of the samples and present in more than 80% of the samples. “Rare” OTUs refer to OTUs with an average relative abundance of <0.1% in all samples. “Moderate” OTUs refer to other OTUs except for rare and abundant OTUs (Galand et al., 2009; Wu et al., 2019; Dai et al., 2022). All further statistical analyses were performed in R (v 4.1.2, http://www.r-project.org).

The β-diversity and its distribution into replacement (β_repl_) and richness difference (β_rich_) components were calculated based on the Jaccard dissimilarity *via* the “adespatial” package in R (Shen et al., 2020). Vertical β-diversity was calculated as the β-diversity between the sum of OTUs in the surface (1 cm) layer and each deeper layer below 1 cm (Wurzbacher et al., 2017; Zhang Y. et al., 2021).

The representative OTUs-based heatmap was to show the similarity of the microbial community across different lake layers and to identify the depth trends. Hellinger transformation of the OTU table and Principal components analysis (PCA) was conducted. To construct the heatmap, representative OTUs were selected with the highest loadings of PC1, PC2, and PC3. The heatmap was created based on Manhattan distance and the clustering algorithm of ward.D using the function “heatmap.2” of the “gplots” package in R. Nonmetric multidimensional scaling (NMDS) was constructed to display differences in microbial communities among different lakes and depths and was plotted using the “phyloseq” package based on Bray–Curtis distance.

The normalized stochasticity ratio (NST) was used to estimate the determinacy and stochasticity of the microbial assembly processes in two vertical lake sediments (Ning et al., 2019). The NST index was calculated based on the null-model analysis, with 50% as the boundary point between more deterministic (<50%) and more stochastic (>50%) assembly processes (Ning et al., 2019). A canonical analysis of principal coordinates (CAP), also known as distance-based redundancy analysis (db-RDA), was used to explain further the relationship between environmental factors and microbial communities in the two lakes. Besides, the relationship between environmental factors and vertical β-diversity and its components (replacement and richness) was analyzed by CAP. CAP was plotted based on Bray–Curtis distance using the “capscale” function of the “vegan” package. Spearman correlation analysis was performed to show the relationship between environmental factors and alpha diversity of microbial communities using the function “corr.test” of the “psych” package in R.

The function annotation of the soil bacterial taxonomy was evaluated based on the Functional Annotation of Prokaryotic Taxa Database (Louca et al., 2016). The top 20 functional groups with the highest proportions of OTUs were visualized using the function “heatmap” in the software Tbtools (Chen et al., 2020). To analyze the depth variation of microbial co-occurrence patterns, networks were constructed for two depth groups or all lake samples based on OTUs present in more than 30% of samples (Dai et al., 2022). The correlations of OTUs were calculated using Spearman’s rank-based correlations, and only highly significant correlations between OTUs were retained (correlation coefficient *ρ* > 0.7 and *ρ* < −0.7, *p*-value <0.05) in the network. The network was performed by the “igraph” package in R (Hartvigsen, 2011) and visualized using the interactive platform Gephi (Bastian et al., 2009).

## Results

### Environmental factors of sediments in two lakes

**Figure 1:**
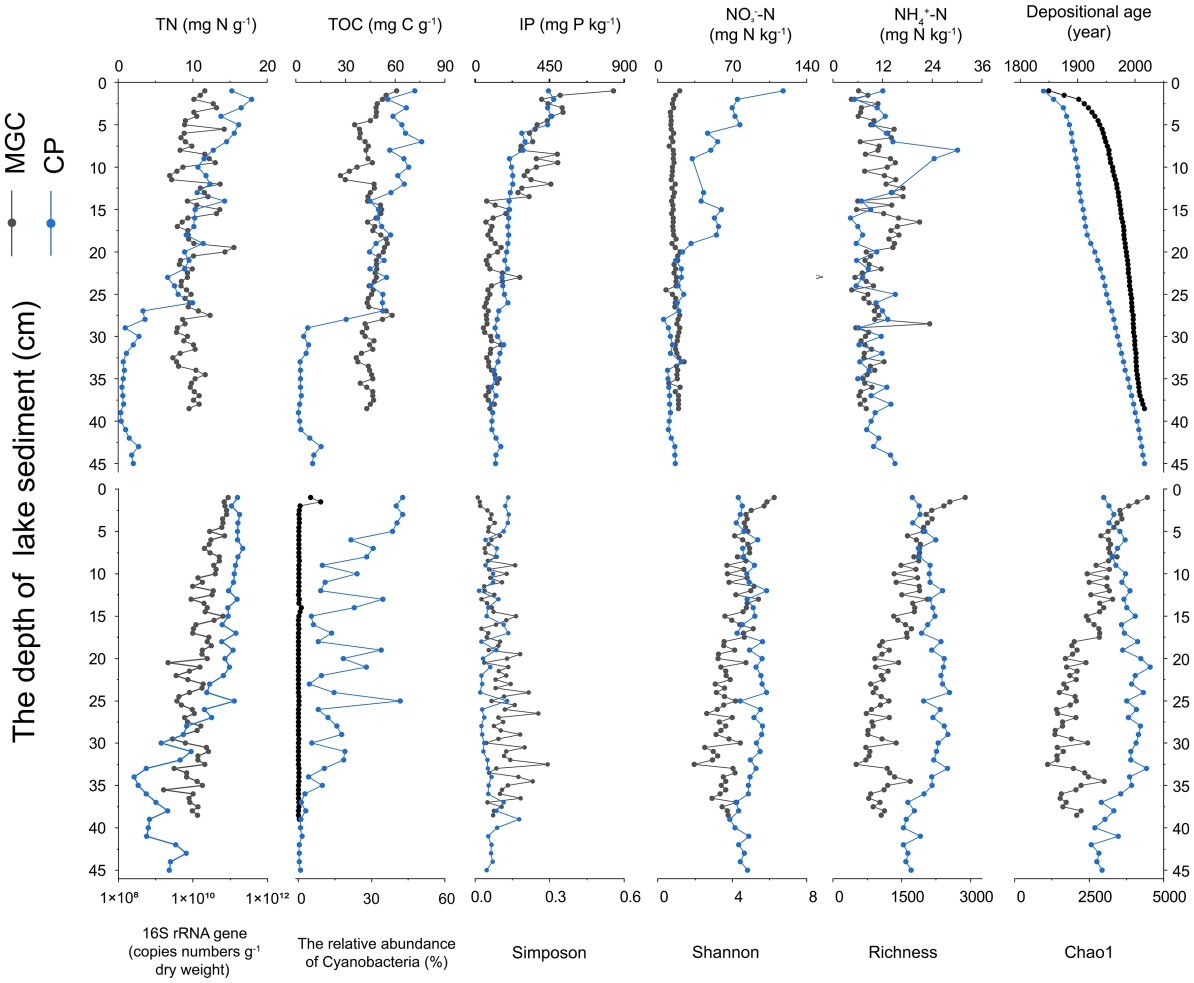
Depth profiles of key physicochemical characteristics (TOC, TN, IP, NO_3_^−^-N, NH_4_^+^-N, and depositional age) and microbial characteristics (copy numbers of 16S rRNA genes), the relative abundance of Cyanobacteria, and alpha diversity indices (Chao1, Richness, Shannon, and Simpson).

Depth distributions of the physicochemical properties in the sediment of two lakes are shown in Figure 1. The results showed that the IP contents in lake MGC decreased with depth, ranging from 836.5 to 49.5 mg kg^−1^, while TN, TOC, NO-3-N, and NH+ 4-N contents did not directly correlate with depth. In lake CP, TN and TOC contents decreased with depth and dropped sharply at 25–30 cm, from 9.97 to 0.94 mg g^−1^ and 52.50 to 4.60 mg g^−1^, respectively. IP and NO-3-N contents were also decreased with depth, while NH+ 4-N content was not significantly correlated with depth. The ^210^Pb dating indicated that the sedimentary cores in both lakes were deposited over the past *ca.* 167 and 176 years (Figure 1).

### Alpha-diversity and abundance of bacteria varied with depth

The abundance of bacteria in the sediments of both lakes decreased with depth (Figure 1), as quantified using the qPCR method. The copy numbers of 16S rRNA genes in lake CP ranged from 2.65 × 10^8^ to 2.21 × 10^11^ g^−1^ dry soil, while in lake MGC ranged from 1.61 × 10^9^ to 8.96 × 10^10^ g^−1^ dry soil.

Alpha diversity indices, including Chao1, Richness, Simpson, and Shannon, were calculated based on the normalized OTU table (Figure 1). Lake CP had higher Chao1, Richness, and Shannon indices than lake MGC. Simpson indices were the opposite. The Chao1, Richness, and Shannon indices all negatively correlated with depth in lake MGC (*p* < 0.001, Supplementary Table S1). Conversely, the Simpson indices increased with depth (*p* < 0.001, Supplementary Table S1). The Chao1, Richness, and Shannon indices in lake CP remained constant or very slightly increased with depth between 1 and 35 cm and declined below 35 cm.

Moreover, Spearman correlation analysis showed the association between alpha diversity and environmental factors (Supplementary Table S1), with Chao1 and Shannon indices negatively correlated with depositional age and NO− 3-N (*p* < 0.001) In lake MGC, and positively correlated with TN and IP (*p* < 0.05). The Simpson index in lake MGC showed an opposite relationship with environmental factors. In lake CP, the correlation between the four alpha diversity indices and environmental factors was not significant (*p* > 0.05).

### Bacterial community composition in phylum- and class-level

**Figure 2:**
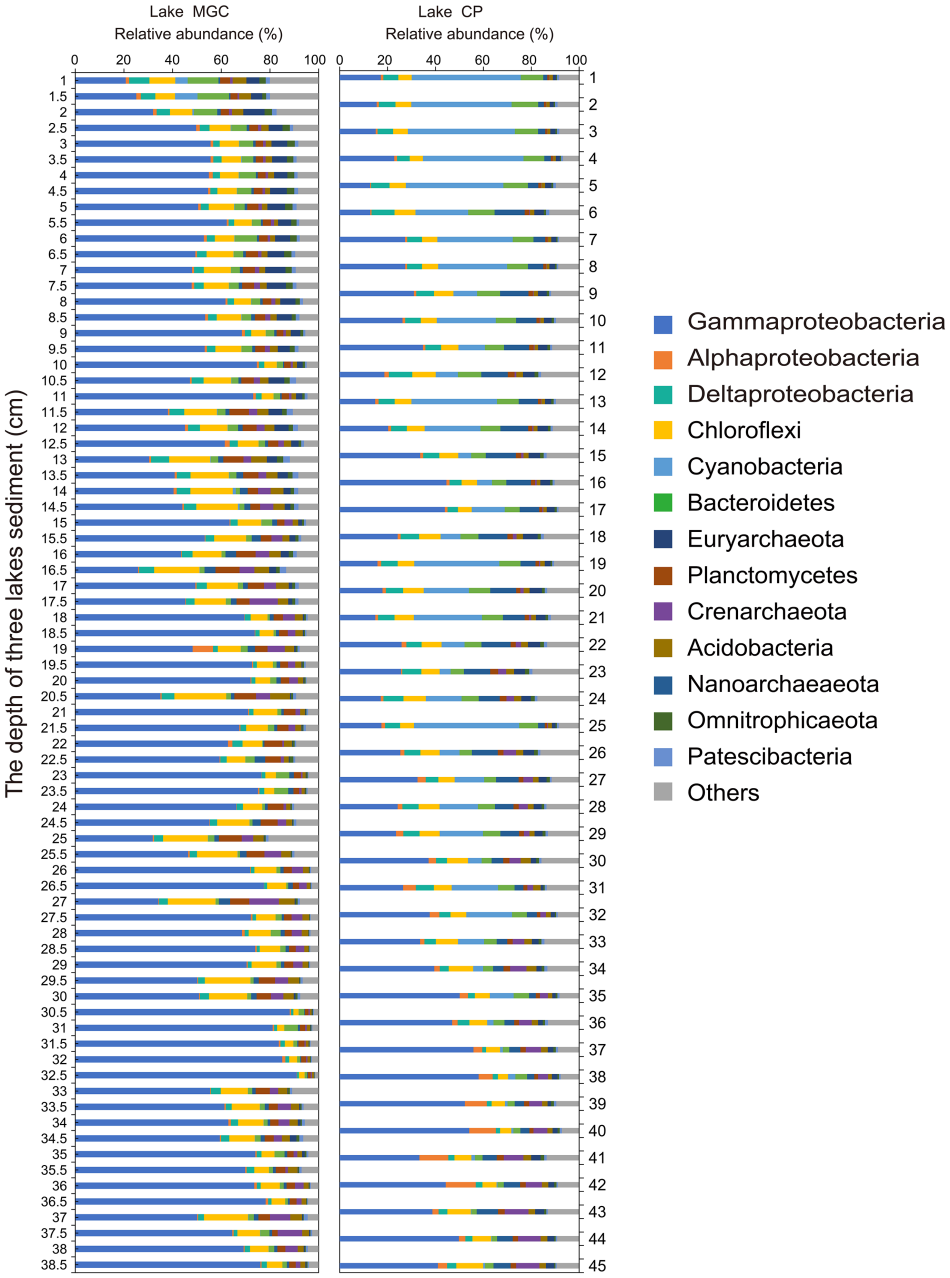
The relative abundance of dominant phyla in sediment cores. Only phyla with an average relative abundance >1% were shown.

Operational taxonomic units were classified into 65 bacterial phyla in sediments. The phyla with relative abundance higher than 1% in the sediments of both lakes were regarded as the abundant phyla (Figure 2). *Proteobacteria* was the most abundant phylum in both lakes, accounting for 62.00 and 38.42% of bacteria in the MGC and CP lakes, respectively. *Gammaproteobacteria* was the largest bacterial class in both lakes, accounting for 57.99 and 30.07% in MGC and CP, respectively. In MGC, *Chloroflexi* (9.74%) was the second most abundant phylum, followed by *Planctomycetes* (4.35%) and *Bacteroidetes* (3.10%). In contrast, *Cyanobacteria* (15.79%) was the second most abundant phylum in lake CP, followed by *Chloroflexi* (7.23%) and *Bacteroidetes* (6.49%).

In lake MGC, the relative abundance of *Gammaproteobacteria* increased with depth (Supplementary Figure S2, *p* < 0.001). *Cyanobacteria*, *Bacteroidetes*, and *Deltaproteobacteria* were enriched in shallow layers, and their relative abundance declined with depth (Supplementary Figure S2, *p* < 0.001). In lake CP, the relative abundance of *Cyanobacteria* was much higher than that in lake MGC, even accounting for 8.30% (average relative abundance) below 25 cm in CP lake.

We separated the OTUs into abundant, moderate, and rare OTUs. The abundant OTUs mainly belonged to *Proteobacteria* in lake MGC (37.74%) and CP (33.44%). The relative abundance of the abundant OTUs in total reads was high, exceeding 60% in each lake (Supplementary Figure S3). However, the number of these abundant OTUs accounts for less than 1% of the total OTUs (Supplementary Figure S3).

### The β-diversity of vertical sediments

**Figure 3:**
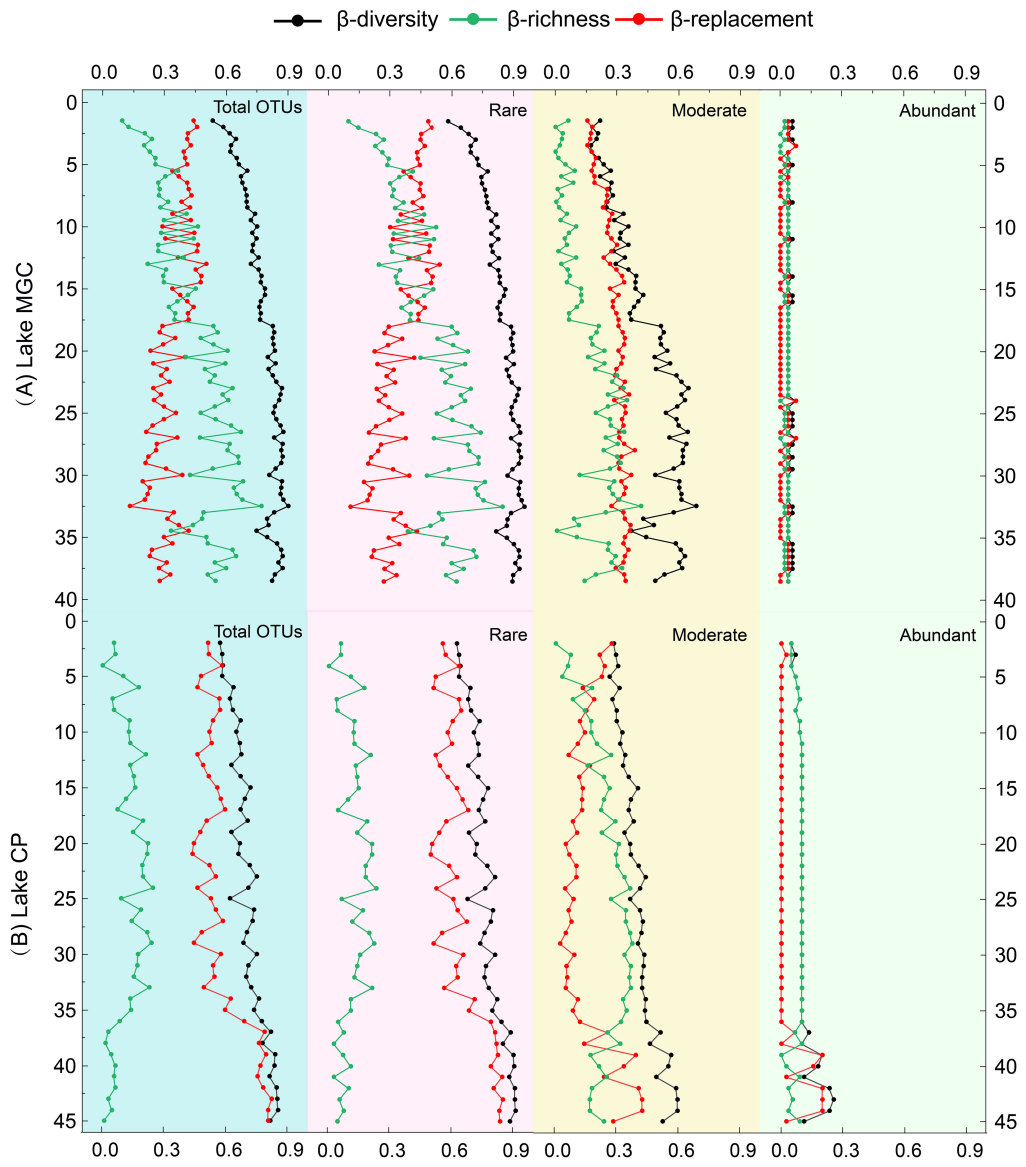
Vertical β-diversity and its components (replacement and richness) based on the total OTUs and partial OTUs (abundant OTUs, moderate OTUs, and rare OTUs).

The total β-diversity increased with depth, between 0.62–0.90 and 0.57–0.85 in MGC and CP lakes, respectively, (Figure 3). In lake MGC, species replacement (β_repl_) generally decreased with depth, and richness differences (β_rich_) increased with depth (Figure 3). Conversely, in lake CP, species replacement (β_repl_) was consistently high and increased with depth (35–45 cm) in the deep layer, and richness differences (β_rich_) were the opposite, which remained at a lower level and decreased with depth at 35–45 cm (Figure 3). The trend in β-diversity and its components (β_repl_ and β_rich_) of the rare OTUs was consistent with the entire community in each lake. In contrast, the β-diversity and its components (β_repl_ and β_rich_) of abundant OTUs remained stable and were consistently low in both lakes.

The heatmap analysis based on representative OTUs showed that the microbial communities in each lake had a depth-related trend (Supplementary Figure S4), with samples from deeper layer being separated from shallow layers. In the MGC lake, samples from various layers were divided into shallow layers (1–17.5 cm) and deep layers (18–38.5 cm). The heatmap of lake CP showed that the samples of shallow layers (1–22 cm) were separated from samples of deep layers (23–45 cm). The OTUs in class *Gammaproteobacteria* were abundant in both shallow and deep layers of MGC. However, the different OTUs in *Gammaproteobacteria* have different depth-related distributions. OTU4, OTU469, and OTU14 were only abundant in the deep layer, while OTU2, OTU17, and OTU18 were enriched in both shallow and deep layers. The depth distribution of *Gammaproteobacteria* OTUs in lake CP was consistent with that in lake MGC. In addition, OTUs belonging to *Cyanobacteria*, which include OTU7 and OTU43, have a higher relative abundance in the shallow layers (1–22 cm) of lake CP.

Moreover, NMDS revealed the difference in microbial community structure among samples with different lakes and depths (Supplementary Figure S5). There was a clear separation of microbial community composition between lake MGC and lake CP by NMDS (*R* = 0.72, *p* = 0.001). Furthermore, depth-related trends of microbial communities were observed in these two lakes (*R* = 0.15, *p* = 0.001).

### Potential function prediction of microbial community

**Figure 4:**
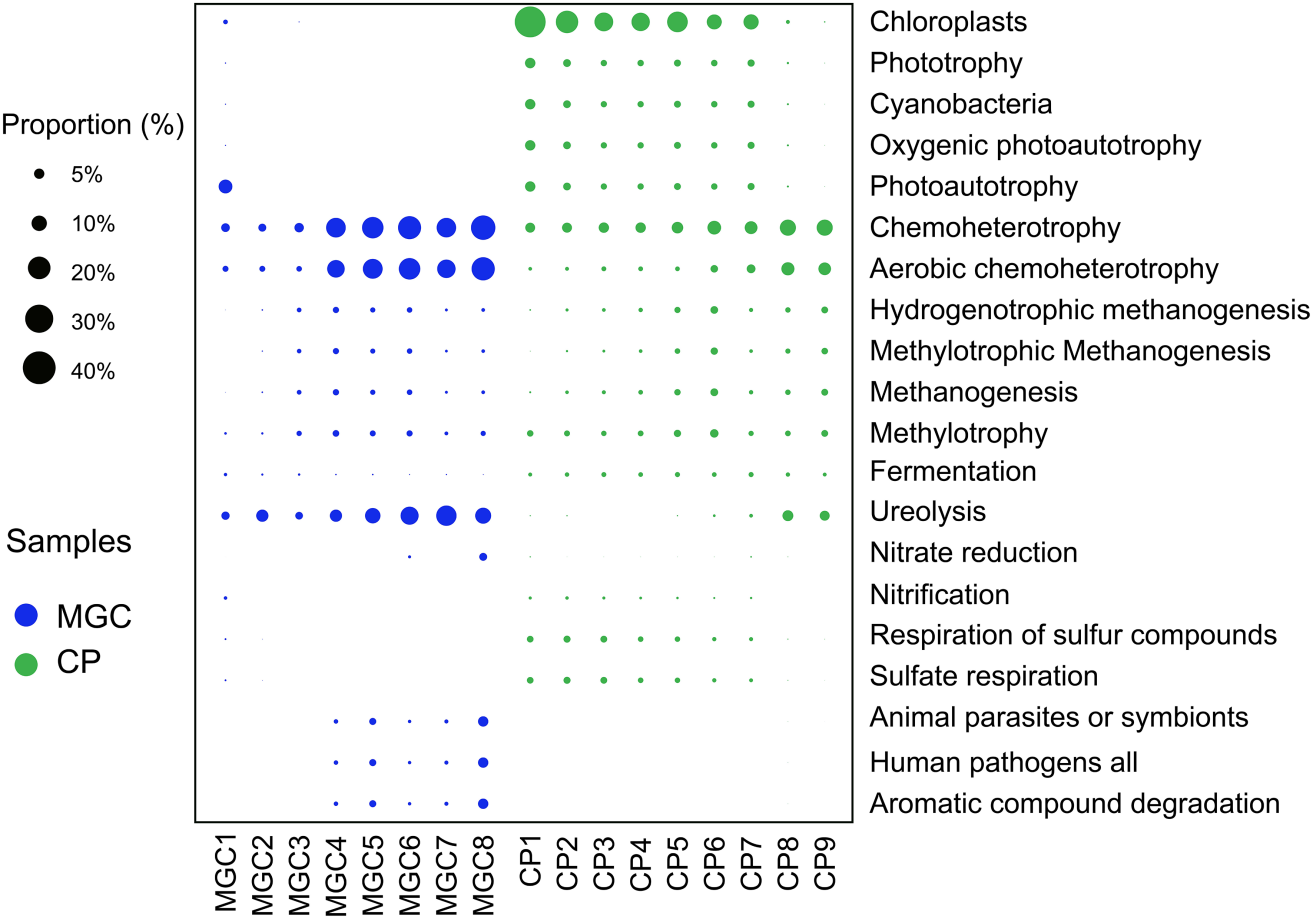
Predicted functional groups of soil microbial community. Only the top 20 functional groups were shown in the plot. The relative abundances of the functional groups were indicated by bubble size.

Potential function prediction was calculated by FAPROTAX analysis. A total of 1,184 OTUs (9.29% of all OTUs) were assigned to 59 functional groups. The results of the predictions showed that the potential functions varied and changed with the sediment depth (Figure 4).

The functions involved in the carbon cycle were the primary and abundant functions. In lake MGC, photoautotrophy was abundant in surface sediments (1–5 cm), while chloroplast was most abundant in shallow sediments (1–25 cm) of lake CP. Chemoheterotrophy, aerobic chemoheterotrophy, and ureolysis were the main functions in deeper sediments of lake MGC (15–38.5 cm) and lake CP (30–45 cm). In the sediments below 10 cm, the functional groups involved in methanogenesis were more abundant. In addition, for the functions involved in the nitrogen cycle, nitrification was active in the surface sediments (1–5 cm). In comparison, nitrate reduction was more abundant in the deep sediments below 35 cm in lake MGC. In the sediments above 25 cm in lake CP, the functional groups involved in the sulfur cycle, such as the respiration of sulfur compounds and sulfite respiration, were more abundant (Figure 4).

### Correlation between microbial community and vertical β-diversity with environmental factors

The CAP was performed to illustrate the influence of environmental factors on microbial communities and vertical β-diversity (Supplementary Figure S6). All environmental factors, including TN, TC, IP, NO− 3-N, and NH+ 4-N, depth and depositional age, accounted for 18.92 and 62.42% of the total variations for microbial communities in lake MGC and CP, respectively, in Supplementary Figure S6A. CAP1 and CAP2 are the first two constrained axes in CAP coordinates, which account for 52.73 and 18.22% of the variation for lake MGC and 78.54 and 15.56% of the variation for lake CP, respectively. This result was consistent with NST, which indicated that the community assembly of lake CP (NST = 46.41%) was more deterministic than that of lake MGC (NST = 75.57%). For the single environmental factor, depth was found to be the most critical factor in both lakes, accounting for a significant portion of the total variation.

The CAP results showed that environmental factors accounted for 68.05 and 81.93% of the total variations in β-diversity in MGC and CP, respectively, (Supplementary Figure S6B). Depth is the most important factor, contributing 60.74 and 48.80% of the variation in β-diversity in MGC and CP (*p* < 0.001). In MGC, the second crucial variable was IP contents, which explained 53.83% of the variation (*p* < 0.001), while in CP, depositional age and TOC were the second and third crucial variables, explaining 46.17 and 36.92% of the variation, respectively. In terms of the relationship between replacement and richness with environmental factors, the results differ between MGC and CP. In MGC, replacement increased with IP but decreased with depth and NO− 3-N, while richness showed the opposite trend. In CP, replacement increased with depth but decreased with TOC, TN, NO− 3-N, and IP, and richness had a weak correlation with these environmental factors.

### Co-occurrence network analysis

Networks were built based on all samples in each lake to explore co-occurrence patterns among the microbial community (Supplementary Figure S7). The network of lake MGC consisted of 522 nodes and 10,293 links, with main modules represented in different colors. Modules 1 and 2 had high relative abundance in shallow sediments (1–17.5 cm) and mainly consisted of *Chloroflexi* and *Nanoarchaeaeota*. The microbial community of module 3 was enriched in deep sediments (18–38.5 cm) and mainly consisted of *Chloroflexi* and *Acidobacteria*. Besides, in modules 1 and 4, the abundant class *Gammaproteobacteria* was enriched in deep sediments (18–38.5 cm). In the network of lake CP, there are 1,173 nodes and 47,402 links. Modules 1 and 4 had higher abundance in deep sediments (23–45 cm). *Gammaproteobacteria* was abundant in module 1, while *Euryarchaea* were enriched in module 4. Module 2 mainly consisted of the phylum Cyanobacteria, which was enriched in shallow sediments (1–22 cm).

**Figure 5:**
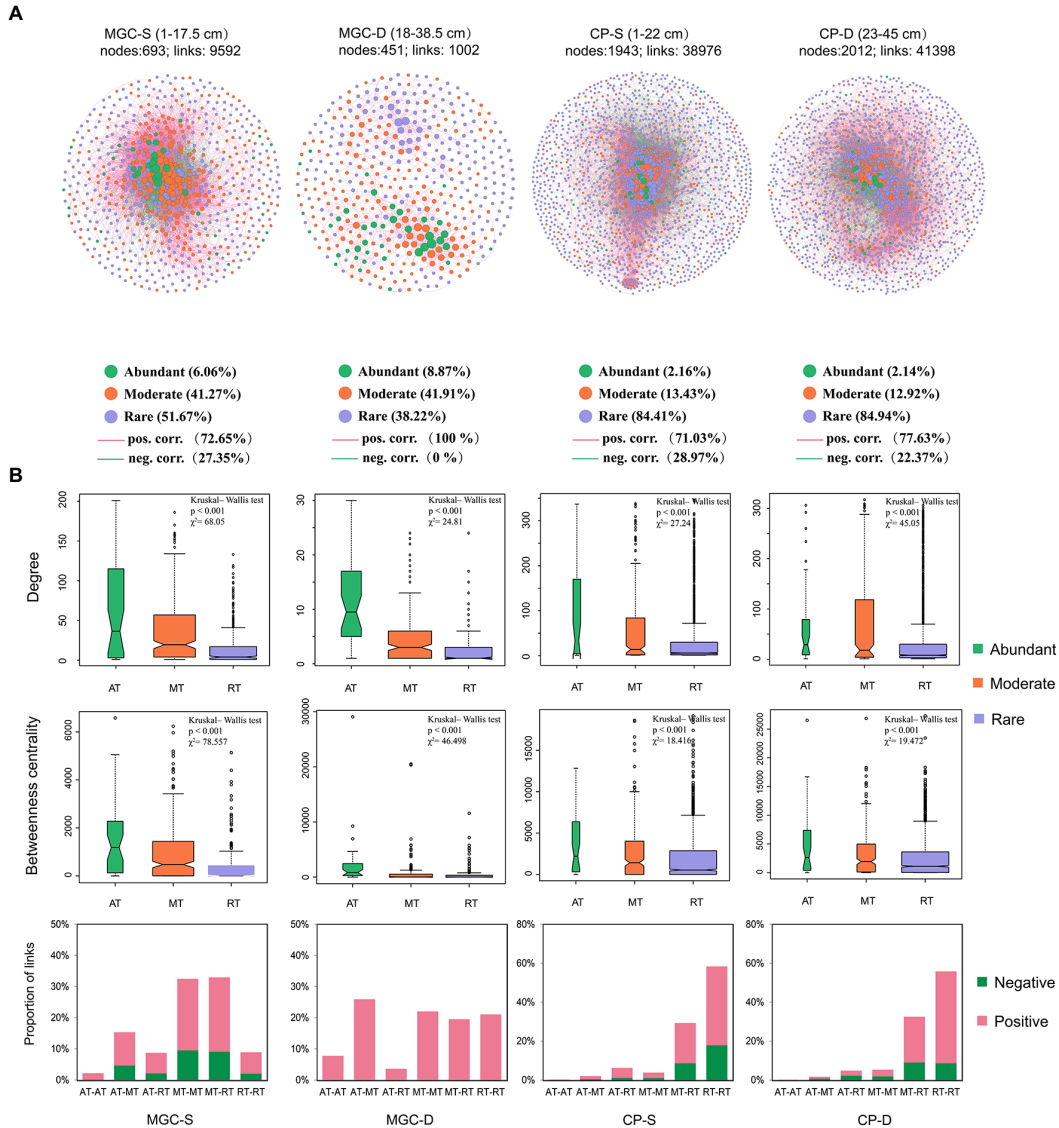
The co-occurrence network and its topological properties based on OTUs of each depth group in lake sediment. **(A)** The networks for different depth groups. The network name, e.g., MGC-S and MGC-D, S and D represent the shallow and deep depth group, respectively. Network node represents a bacterial taxon at the OTU level, and the size of the node represents the “degree” (the number of connections). Nodes are denoted by different colors representing their affiliated taxa (abundant taxa, moderate taxa, and rare taxa). The significant spearman correlations between OTUs are retained (correlation coefficient *ρ* > 0.7 and <−0.7, *p* < 0.05). The red and green edges signify positive and negative correlations, respectively. **(B)** The topological properties of the network, including nodes’ degree, betweenness centrality, and proportion of network links. The difference in nodes’ degree and betweenness centrality level within different taxa were tested by Kruskal–Wallis test. The proportion of network links was shown by the affiliated taxa of the associated OTUs. AT, abundant taxa; MT, moderate taxa; RT, rare taxa.

The networks of the microbial community in surface and deep sediments were established for each lake to analyze the co-occurrence patterns (Figure 5). The network topology indices are shown in Supplementary Table S2. The networks from different depth layers showed that both rare and moderate OTUs accounted for a high proportion in both lakes (Figure 5A). The results showed that the degree and betweenness centrality of abundant OTUs were significantly higher compared to rare and moderate OTUs (Figure 5B, *p* < 0.001, Kruskal–Wallis test). The positive and negative interactions among OTUs were also calculated, with a decrease in the proportion of negative interactions observed with increasing depth. Furthermore, negative interactions mainly existed in moderate/rare OTUs or between rare and moderate OTUs (Figure 5B).

## Discussion

### Vertical variation of microbial community composition in two lakes

Microbial communities in two lake sediments varied with the depth due to the depth gradient of biogeochemical properties, which provided specific niches for microorganisms with diverse metabolic capabilities (Wilms et al., 2006). The microbial community can be grouped into different clusters based on depth (Supplementary Figures S4, S7), a phenomenon that has been observed in other lake sediments (Wurzbacher et al., 2017). The shallow layer clusters mainly consist of the phyla *Proteobacteria*, *Cyanobacteria* and *Bacteroidetes*. *Proteobacteria* are commonly abundant in lake sediments, and the composition of their significant classes and their proportions varies with lakes (Zhang et al., 2015). In lake CP, *Cyanobacteria* was consistently abundant at depth of 1–35 cm. Previous studies have noted the predominance of *Cyanobacteria* in surface sediment, which may be related to cyanobacterial blooms (Latour et al., 2007; Liu et al., 2010). Lake CP was oligotrophic before the 1880s, but its nutrient conditions improved over time, which may have contributed to the increased growth of algae (Jiang et al., 2020). *Cyanobacteria* can also adapt to the cold, dark, and anoxic deep sediment, possibly due to its cold-shock DEAD-box protein A, which has been shown to play a role in cyanobacterial adaptation to low temperatures (Chamot et al., 1999; Redder et al., 2015). *Bacteroidetes*, which are chemoorganotrophic bacteria with phototrophic capabilities, are also commonly found in surface lake sediments (Newton et al., 2011) and positively correlated with DOC concentration (Eiler et al., 2003), likely explaining their predominance in shallow layers.

### Vertical variation of β-diversity and its components in lake sediments

Exploring the β-diversity and its components (replacement and richness) can provide insights into the processes driving the vertical variation of microbial communities. In this study, the richness effect constantly increased with depth while the replacement effect decreased with depth in lake MGC (Figure 3). This trend reflected that the microbial community in the deep layer was a subgroup of the surface microbial community, with the community shifting from a complicated and rich community at the surface to a more atrophic community in the deeper sediment. This transition was mainly driven by the filter effect, which might result from abiotic limitations, such as nutrient reductions and space shrinking (Hu et al., 2012). In contrast, the trend of replacement and richness effect in lake CP was the opposite. The richness effect maintained a low level, while the replacement effect was constantly abundant and increased with depth (Figure 3). This trend of increased replacement effects in the deep layer has also been found in marine sediment (Zhang Y. et al., 2021), which may indicate the existence of an inactive seed bank with a great variety in the deep layer. The trend of β-diversity components in lake CP was consistent with the microbial community structural model that proposed niche specialists adapted to the specific environmental conditions at different depths of layers (Wurzbacher et al., 2017). During the burial process for some lake sediments, the different patterns of environment parameters with depth may result in different β-diversity portioning patterns in vertical sediments. Our results suggested that most environmental factors in MGC were not depth-related, and only IP contents decreased with increasing depth. IP was found to play a critical role in shaping the β-diversity pattern in MGC. While in CP, most depth-related environmental factors such as TOC and TN had a significant impact on the β-diversity portioning pattern. Furthermore, spatial and biotic factors also influenced the β-diversity portioning patterns in lake sediments (Wu et al., 2020). Further research can be conducted in the future by incorporating more parameters to further explore these patterns.

### The co-occurrence network of microbial interactions at various depths of lake sediments

In this study, the proportion of negative correlation among OTUs in the network decreased with sediment depth in both MGC and CP (Figure 5). Resource competition and metabolic cross-feeding are the main drivers of the microbial community assembly (Machado et al., 2021). The interactions between microbial communities are significantly influenced by the availability of nutrients (Gulis and Suberkropp, 2003; West et al., 2007). Most microorganisms are auxotrophic and require external nutrients for growth. In a nutrient-rich environment, active microbial growth may result in competition for necessary nutrients and the inhibition of the growth of competitor growth in the community (Ratzke et al., 2020). In comparison, collaboration may encourage microbial colonization and growth in low-nutrient conditions, facilitating adaptation to oligotrophic environments (Velez et al., 2018). Lake sediments can provide microbes with different nutrient environments at various depths, such as decreasing availability of electron acceptors and substrates with depth. Consequently, negative microbial interactions decreased with depth due to the reduction of major nutrients including IP in MGC and TOC, TN, and IP in CP. This phenomenon was also found in marine and other lake sediments (Dai et al., 2022; Zhang et al., 2022), where negative microbial interactions were observed to be more prevalent in rich-nutrient conditions, and positive interactions were more predominant in low-nutrient conditions.

Our analysis also revealed that the number of rare OTUs accounted for a higher proportion of total nodes, and that most negative correlations existed among moderate or rare OTUs. This suggested that rare and moderate OTUs have more overlapping ecological niches. Besides, abundant OTUs had higher degree and betweenness centrality levels, which aligns with previous findings (Zhang et al., 2020, Zhang Y. et al, 2021), indicating that abundant OTUs play a critical role in maintaining the structure of microbial interactions in the network.

## Conclusion

In this study, we conducted a comprehensive analysis of the microbial community composition, diversity, and interaction in sediment samples collected from two lakes on the Tibetan plateau. Our findings demonstrate the significance of both abundant and rare taxa in shaping the β-diversity and microbial interactions. Our results reveal similarities in microbial community compositions at the phylum level between the two lakes, with *Cyanobacteria* being more prevalent in lake CP. Additionally, we observed varying β-diversity patterns correlated with environmental factors. Our results indicate that the pattern of microbial interactions varied with depth, with evidence suggesting that higher nutrient levels may lead to negative interactions among microbes, while positive interactions are more prevalent in low-nutrient environments. These results broaden our understanding of patterns of β-diversity partitioning and microbial interactions in vertical sediment samples on the Tibetan plateau and provide new insights into mechanisms driving microbial community variation.

## Data availability statement

The datasets presented in this study can be found in online repositories. The names of the repository/repositories and accession number(s) can be found at: https://www.ncbi.nlm.nih.gov/, PRJNA876099.

## Author contributions

YD, TH, and CheH designed the study. TH and ZZ sampled the sediments. TH and CheH were responsible for microbial lab work. XZ and YD analyzed the data, prepared figures, and wrote the initial manuscript. LC, KL, YL, and ChaH reviewed and edited the manuscript. All authors contributed to the article and approved the submitted version.

## Funding

This research was funded by the National Natural Science Foundation of China (Grant No. 41971077) and the Second Tibetan Plateau Scientific Expedition and Research (STEP) program (Grant No. 2019QZKK0503).

## Conflict of interest

The authors declare that the research was conducted without any commercial or financial relationships that could be construed as a potential conflict of interest.

## Publisher’s note

All claims expressed in this article are solely those of the authors and do not necessarily represent those of their affiliated organizations, or those of the publisher, the editors and the reviewers. Any product that may be evaluated in this article, or claim that may be made by its manufacturer, is not guaranteed or endorsed by the publisher.
