# Supplementary material for: Vertical variations in microbial diversity, composition, and interactions in freshwater lake sediments on the Tibetan plateau
Source: Front Microbiol. 2023 Mar 8;14:1118892. doi: 10.3389/fmicb.2023.1118892 (PMC10031068; doi:10.3389/fmicb.2023.1118892)
Supplement: Supplementary file 1 [file Data_Sheet_1.docx]

Supporting Information

**The vertical variations in microbial diversity, composition and interactions in freshwater lake sediments on the Tibetan plateau**

Xinshu Zhu^1^, Yongcui Deng^1, 2^^*^, Tao Huang^1, 2*^, Cheng Han^1^, Lei Chen^1^, Zhigang Zhang^1^, Keshao Liu^3^, Yongqin Liu^3, 4^ and Changchun Huang^1, 2^

^1^ School of Geography, Nanjing Normal University, Nanjing 210023, China.

^2^ Jiangsu Center for Collaborative Innovation in Geographical Information Resource Development and Application, Nanjing, 210023, China.

^3^ State Key Laboratory of Tibetan Plateau Earth System, Resources and Environment (TPESRE), Institute of Tibetan Plateau Research, Chinese Academy of Sciences, Beijing 100101, China.

^4^ Center for the Pan-third Pole Environment, Lanzhou University, Lanzhou 730000, China

*** Correspondence:**

Yongcui Deng

Email: [dengyongcui@njnu.edu.cn](mailto:dengyongcui@njnu.edu.cn)

Tao Huang

Email: huangtao198698@126.com

**Table S1**. Spearman correlation analysis of alpha diversity indices (Chao 1, Richness, Simpson, and Shannon) and soil physicochemical properties (**TOC** (Total Organic Carbon), **TN** (Total Nitrogen), **IP** (Inorganic Phosphorus), **NO_3_^-^-N** (Nitrate Nitrogen), **NH_4_^+^-N** (Ammonium Nitrogen).

|  |  | TOC | TN | IP | NO_3_^-^-N | NH_4_^+^-N | Depth | Depositional  age |
| --- | --- | --- | --- | --- | --- | --- | --- | --- |
| Lake MGC | Chao1 | 0.05 | 0.26* | 0.75  *** | -0.56  *** | 0.13 | -0.78  *** | -0.78  *** |
|  | Richness | 0.05 | 0.26* | 0.71  *** | -0.55 | 0.17 | -0.79  *** | -0.79  *** |
|  | Simpson | -0.06 | -0.05 | -0.37 | 0.28  *** | -0.18 | 0.50  *** | 0.50  *** |
|  | Shannon | 0.02 | 0.14 | 0.55  *** | 0.46  *** | 0.19 | -0.70  *** | -0.70  *** |
| Lake CP | Chao1 | -0.01 | -0.01 | 0 | -0.11 | -0.38 | -0.13 | -0.13 |
|  | Richness | 0.06 | 0.06 | 0.06 | -0.07 | -0.37 | -0.19 | -0.19 |
|  | Simpson | 0.12 | 0.18 | 0.22 | 0.33 | 0.10 | -0.19 | -0.19 |
|  | Shannon | 0.05 | 0.03 | -0.01 | -0.15 | -0.24 | -0.06 | -0.06 |

The table shows the correlation coefficients. The stars represent the significance of the correlation. * P< 0.05, ** P< 0.01 and *** P< 0.001.

**Table S2**. Network topology index for co-occurrence networks of the microbial community in each depth group (Figure 5). **MGC-S:** the network based on the shallow depth group (1-17.5 cm) of lake MGC. **MGC-D:** the network based on the deep depth group (18-38.5 cm) of lake MGC. **CP-S:** the network based on the shallow depth group (1-22 cm) of lake CP. **CP-D:** the network based on the deep depth group (23-45 cm) of lake CP.

|  | Average Degree | Weighted Degree | Average  Path length | Density | Modularity | Average Clustering Coefficient |
| --- | --- | --- | --- | --- | --- | --- |
| MGC-S | 27.68 | 41.83 | 3.24 | 0.04 | 0.28 | 0.51 |
| MGC-D | 4.44 | 6.67 | 6.37 | 0.01 | 0.71 | 0.41 |
| CP-S | 40.12 | 61.58 | 3.75 | 0.02 | 0.29 | 0.40 |
| CP-D | 41.15 | 31.60 | 3.75 | 0.02 | 0.31 | 0.37 |

**Figure S1.** Sampling site of lake Mgecuo (MGC) and Cuopu (CP) sediment cores.


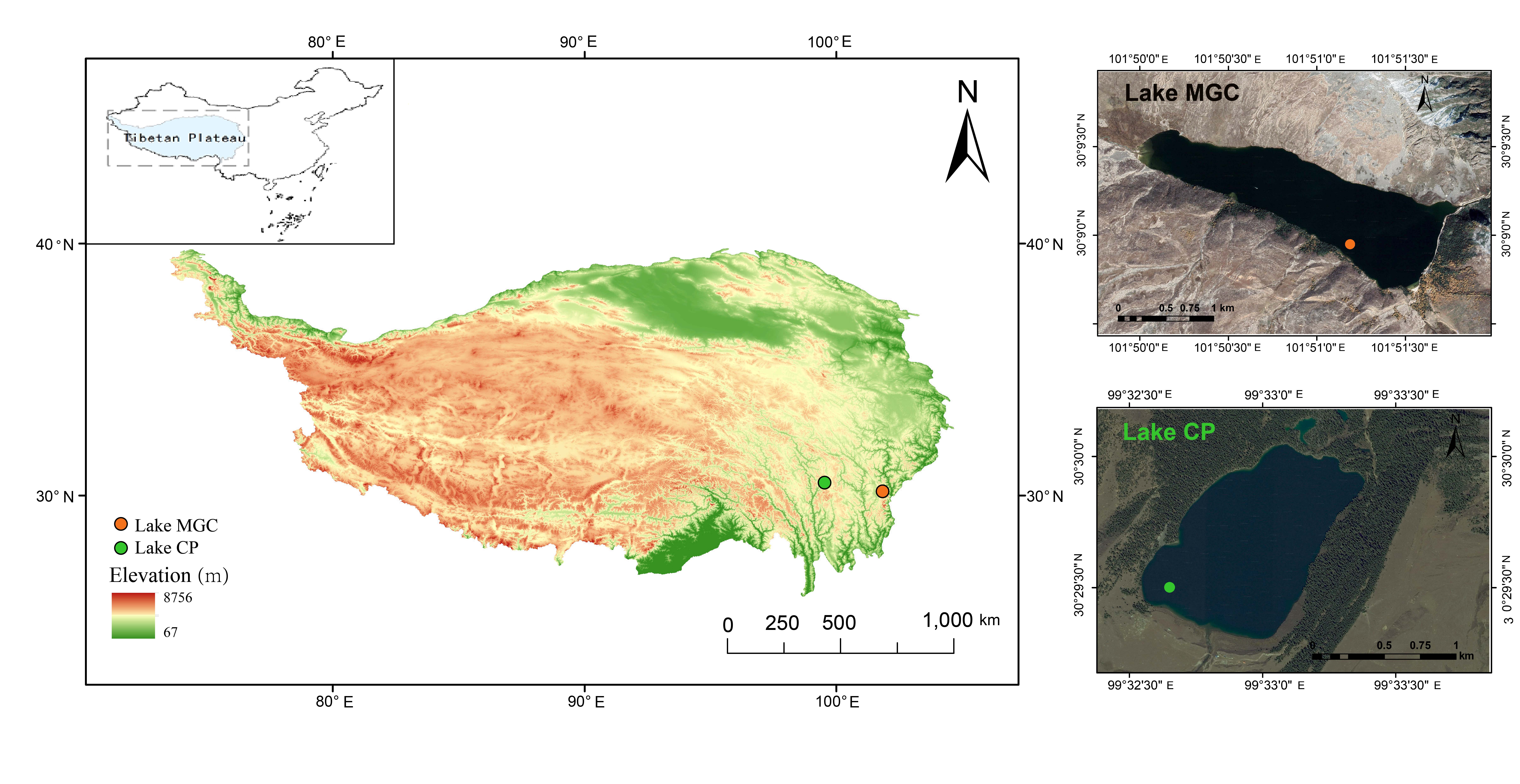


**Figure S2**. The relative abundance of depth-related bacterial phyla. Spearman correlations between depth and relative abundance of bacterial phyla were calculated, and only abundant bacterial phyla (average relative abundance >1%) significantly correlated with depth were retained (P<0.05).


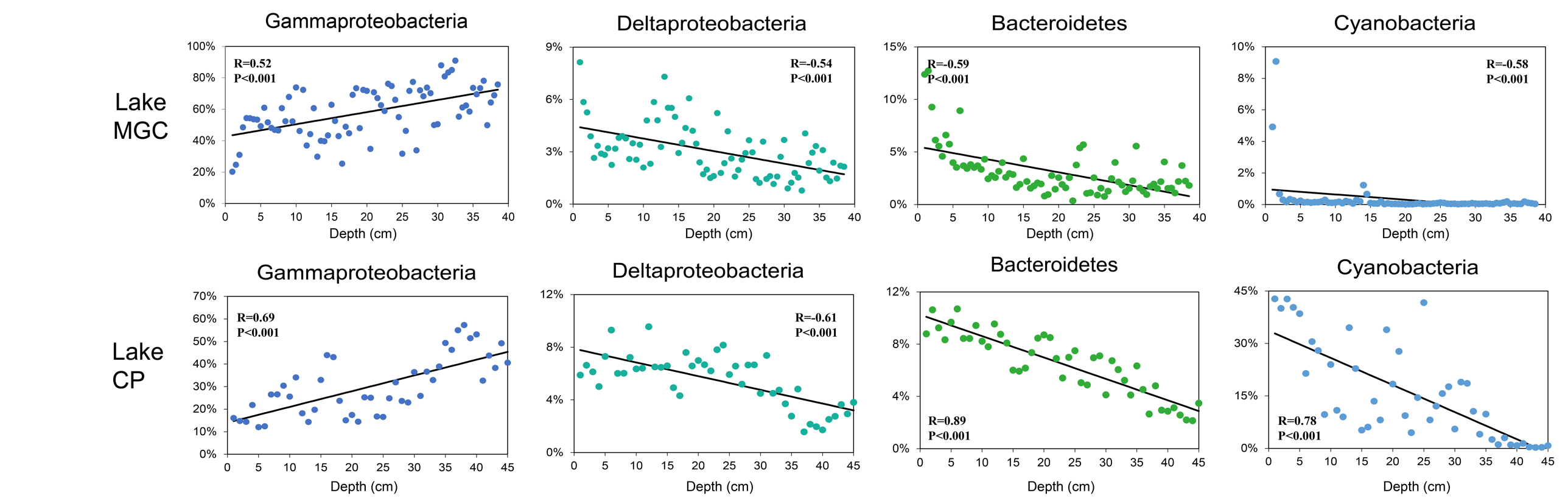


**Figure S3**. The sequence numbers of different taxa (abundant taxa, moderate taxa and rare taxa) and their relative abundance.


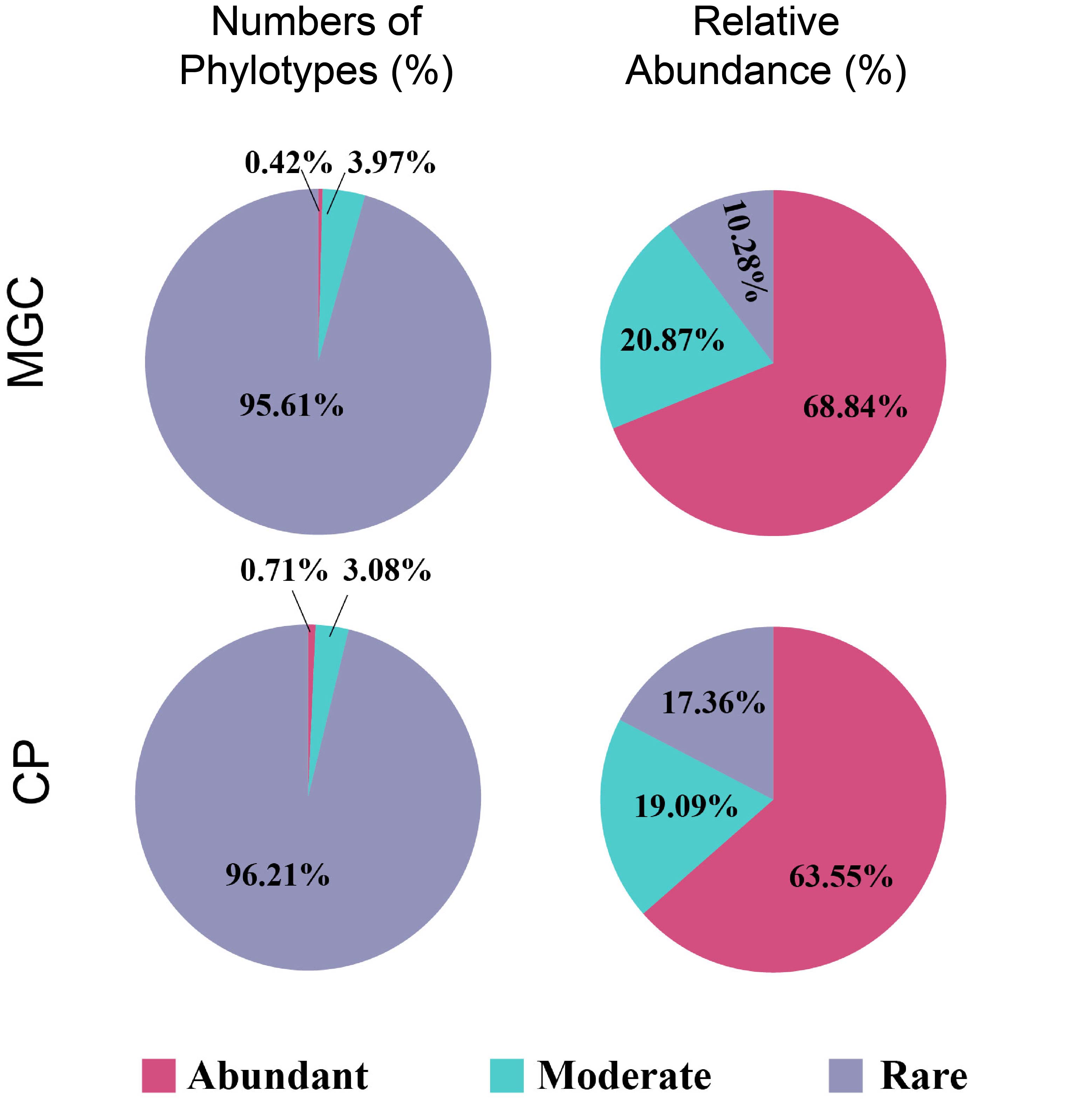


**Figure S4**. Heatmap of relative abundance of selected OTUs in MGC and CP lakes. The samples were clustered according to Manhattan distance. On the right of the heatmap is the taxonomy information for each OTU.





**Figure S5**. Nonmetric multidimensional scaling (NMDS) plot based on Bray distance.


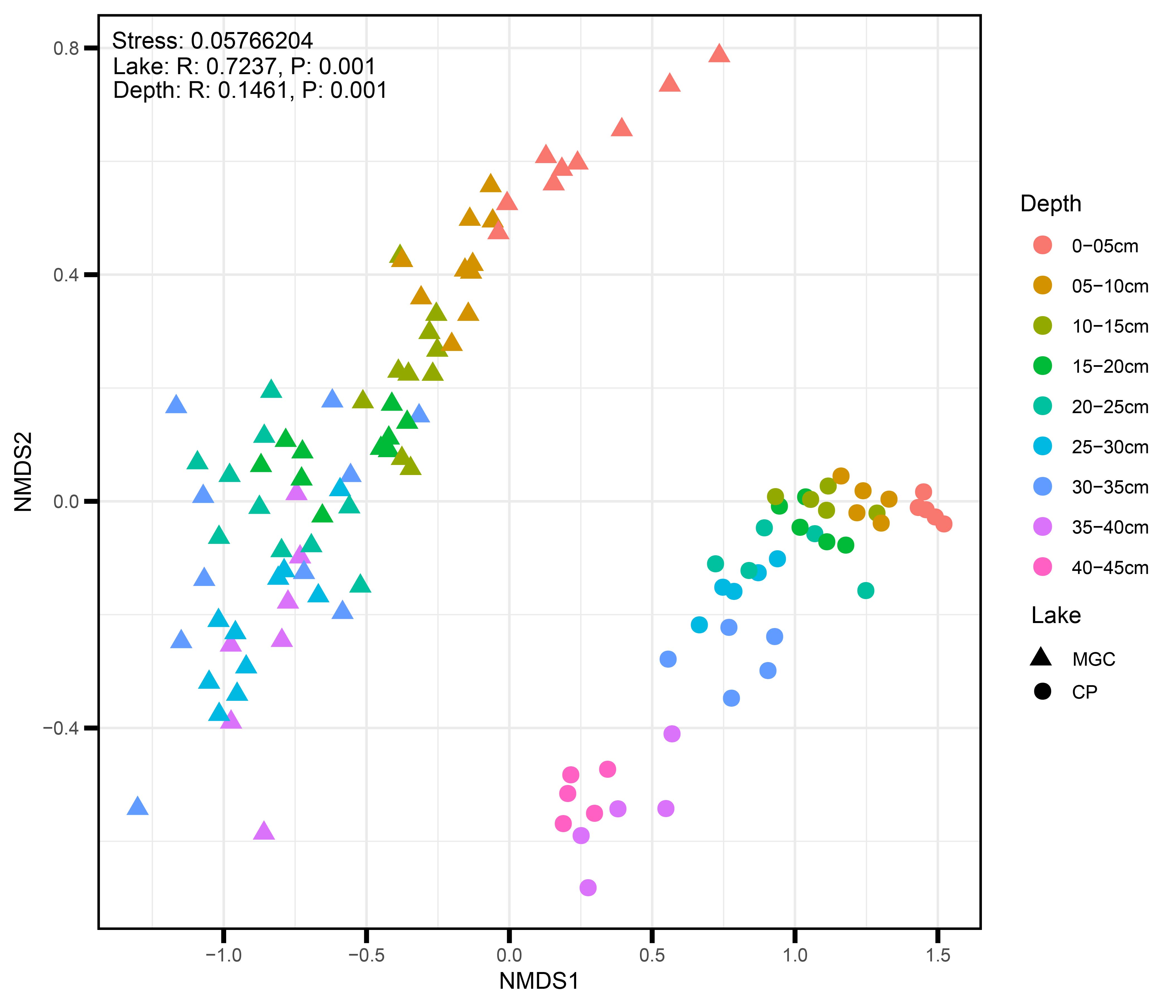


**Figure S6**. Canonical analysis of a principal coordinates (CAP) plot. The CAP plot shows the effect of soil properties (TOC, TN, IP, NO_3_^-^-N, NH_4_^+^-N, depth and depositional age) on (A) microbial communities and (B) vertical β-diversity.


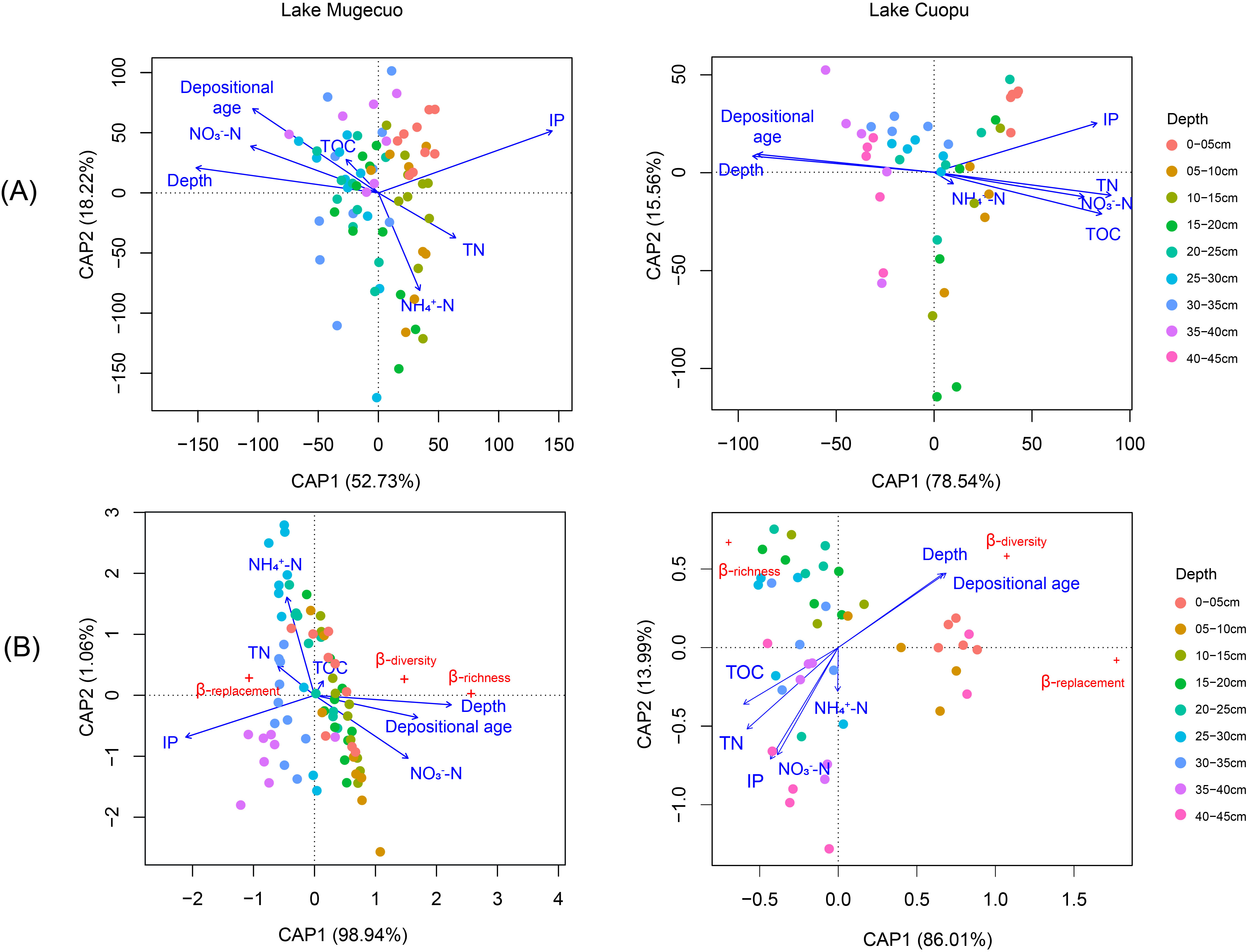


**Figure S7**. Co-occurrence network of the microbial community in all sediment samples and microbial community structure of main modules. (A) Co-occurrence network with OTUs colored by modularity and the top 4 abundant modules. Significant Spearman correlations are retained (correlation coefficient ρ >0.7 and <-0.7, P < 0.05). The size of each node depends on the number of connections. (B) Relative abundance of phyla in the top 4 abundant modules.
